# IDH1 Promotes Foam Cell Formation by Aggravating Macrophage Ferroptosis

**DOI:** 10.3390/biology11101392

**Published:** 2022-09-23

**Authors:** Ben Li, Chufan Wang, Peng Lu, Yumeng Ji, Xufeng Wang, Chaoyang Liu, Xiaohu Lu, Xiaohan Xu, Xiaowei Wang

**Affiliations:** 1 Department of Cardiovascular Surgery, The First Affiliated Hospital, Nanjing Medical University, Nanjing 210000, China; 2 The Friendship Hospital of Ili Kazakh Autonomous Prefecture Ili, Jiangsu Joint Institute of Health, Yining 835000, China

**Keywords:** IDH1, foam cell, macrophage, ferroptosis, NRF2

## Abstract

**Simple Summary:**

In our study, the involvement of IDH1 in atherosclerotic foam cells was explored. Inhibiting macrophage ferroptosis and foam cell formation by knocking down IDH1 is a promising study direction for better understanding the occurrence and progression of atherosclerosis, as well as the treatment targets for atherosclerosis.

**Abstract:**

A distinctive feature of ferroptosis is intracellular iron accumulation and the impairment of antioxidant capacity, resulting in a lethal accumulation of lipid peroxides leading to cell death. This study was conducted to determine whether inhibiting isocitrate dehydrogenase 1 (IDH1) may help to prevent foam cell formation by reducing oxidized low-density lipoprotein (ox-LDL)-induced ferroptosis in macrophages and activating nuclear factor erythroid 2-related factor 2 (NRF2). Gene expression profiling (GSE70126 and GSE70619) revealed 21 significantly different genes, and subsequent bioinformatics research revealed that ferroptosis and IDH1 play essential roles in foam cell production. We also confirmed that ox-LDL elevates macrophage ferroptosis and IDH1 protein levels considerably as compared with controls. Ferrostatin-1 (Fer-1), a ferroptosis inhibitor, reduced ox-LDL-induced elevated Fe^2+^ levels, lipid peroxidation (LPO) buildup, lactate dehydrogenase (LDH) buildup, glutathione (GSH) depletion, glutathione peroxidase 4 (GPX4), ferritin heavy polypeptide 1 (FTH1), and solute carrier family 7 member 11 (SLC7A11) protein downregulation. More crucially, inhibiting IDH1 reduced Fe^2+^ overload, lipid peroxidation, LDH, and glutathione depletion, and elevated GPX4, FTH1, and SLC7A11 protein expression, resulting in a reduction in ox-LDL-induced macrophage ferroptosis. IDH1 inhibition suppressed ox-LDL-induced macrophage damage and apoptosis while raising NRF2 protein levels. We have demonstrated that inhibiting IDH1 reduces ox-LDL-induced ferroptosis and foam cell formation in macrophages, implying that IDH1 may be an important molecule regulating foam cell formation and may be a promising molecular target for the treatment of atherosclerosis.

## 1. Introduction

Atherosclerosis is a chronic inflammatory condition of the artery wall in which macrophages play a significant role [1]. The production of foam cells is a key step in the pathogenesis of atherosclerosis, and inhibiting foam cell formation could be a viable way to prevent atherosclerotic lesions from developing [2]. Lipid peroxy radicals and hydroperoxides produced by free radical oxidation of polyunsaturated fatty acids (PUFAs), known as LPO, play a significant role in atherosclerosis [3,4].

A distinctive feature of ferroptosis is intracellular iron accumulation and the impairment of antioxidant capacity, resulting in a lethal accumulation of lipid peroxides leading to cell death [5]. Many pathogenic processes have been linked to ferroptosis, including cancer, ischemic organ damage, neurological illnesses, autoimmune diseases, and atherosclerosis [6,7]. Advanced human atherosclerotic plaques have lipid peroxidation, intraplaque bleeding, and iron deposition [8]. The mechanism of ferroptosis in atherosclerosis, however, is unknown.

IDH1 is a protease that may oxidize isocitrate to oxalosuccinate, which is then converted to α-ketoglutarate. It is found in the cytoplasm and peroxisomes. In studies, IDH1 mutations have been associated with malignant tumors such as secondary glioblastoma, cholangiocarcinoma, and periosteal cartilage tumors [9]. IDH1 mutations in tumors accelerate lipid reactive oxygen species (ROS) accumulation, leading to glutathione depletion through the downregulation of GPX4 [10]. However, it is unknown whether IDH1 enhances ferroptosis and thus worsens atherosclerosis progression.

In our study, the involvement of IDH1 in atherosclerotic foam cells was explored. Inhibiting macrophage ferroptosis and foam cell formation by knocking down IDH1 is a promising study direction for better understanding the occurrence and progression of atherosclerosis, as well as the treatment targets for atherosclerosis.

## 2. Materials and Methods

### 2.1. Data Mining from the Gene Expression Omnibus (GEO) Database

Gene expression profiles can be searched and downloaded using the GEO database. Appropriate microarray datasets were screened from the GEO database to analyze differential genes between foam cells and macrophages. The GEO database provided the gene expression profiles of GSE70126 and GSE70619. The platforms GPL6885 and GPL1261 provided these RNA profiles. GSE70126 is a genomic profile of foam cell macrophages isolated from fat-fed ApoE null mice and non-foamy macrophages isolated from control mice fed a normal diet. GSE70619 is the expression data from foam cells of apolipoprotein E-deficient mice. GEO2R is an online tool provided by the GEO database, which is mainly used for the differential analysis of expression profile chips. GEO2R was used to research the two groups of samples and find genes that were significantly differentially expressed at *p* < 0.05 and |logFC| > 1 experimental conditions.

### 2.2. Analysis of Functional Enrichment

We used DAVID (https://david.ncifcrf.gov) (accessed on 12 August 2021) and Bioinformatics (http://www.bioinformatics.com.cn) (accessed on 12 August 2021) to perform Gene Ontology (GO) and Kyoto Encyclopedia of Genes and Genomes (KEGG) enrichment analysis on differentially expressed genes (DEGs). Bioinformatics is a free online platform for data analysis and visualization [11]. *p* values less than 0.05 are considered statistically significant.

### 2.3. FerrDb Database

FerrDb is the first manually maintained ferroptosis database, as well as a database of ferroptosis-related disorders. Thus far, the database has extracted and sorted 259 regulated genes, 111 markers, and 95 ferroptosis-related disorders from publications about ferroptosis in the PubMed database [12].

### 2.4. Cells and Reagents

The cell bank of the Chinese Academy of Sciences provided RAW264.7 mouse macrophages. Ox-LDL was provided by Guangzhou Yiyuan Biotechnology Co., Ltd., YB-002, Guangzhou, China. Proteintech (Wuhan, China) provided the primary antibodies against IDH1, NRF2, GPX4, SLC7A11, and GAPDH. Abcam (Cambridge, UK) provided the primary antibodies against FTH1. Fer-1 is a potent inhibitor of ferroptosis. Fer-1 was obtained from Sigma-Aldrich (St. Louis, MO, USA).

### 2.5. Treatment and Transfection of Cells

RAW264.7 macrophages were cultivated at 37 °C in 5% CO_2_ using DMEM containing 10% fetal bovine serum (FBS). We divided cells into the following groups at random: control, ox-LDL, ox-LDL + si-IDH1, ox-LDL + Fer-1, ox-LDL + si-IDH1 + Fer-1. RAW264.7 cells were pretreated with 5 µmol/L Fer-1 (24 h), followed by stimulation of ox-LDL for 24 h. Genechem (Shanghai, China) designed and synthesized LV-Idh1-RNAi to target IDH1. CON313 was used as a control, transfected with shRNA to reduce IDH1 levels (targeting sequence: CCGGGCCAAATTAGCTCAGGCCAAACTCGAGTTTGGCCTGAGCTAATTTGGCTTTTTG). To carry out the transfection, we followed the manufacturer’s instructions.

### 2.6. Analysis of Cell Viability

RAW264.7 macrophages were seeded in 96-well plates (3 × 10^3^ cells/well) and treated with several experimental treatments 24 h after cell adhesion, followed by the addition of cell counting kit 8 (CCK-8) reagents (10 µL/well). A microplate reader (Multiskan FC, Waltham, MA, USA) was used to measure the absorbance at 450 nm. Representative images of cell death in each group were collected using an optical microscope (Nikon TS2, Tokyo, Japan).

### 2.7. Western Blotting Analysis

Proteins were separated using 10% SDS-PAGE gels and transferred onto PVDF membranes. After blocking with 5% bovine serum albumin (BSA) for 1 h at room temperature, the membranes were incubated at 4 °C overnight with specific primary antibodies: anti-IDH1 (1:1000, Proteintech, 12332-1-AP), anti-GAPDH (1:1000, Proteintech, 60004-1-Ig), anti-NRF2 (1:1000, Proteintech, 16396-1-AP), anti-GPX4 (1:1000, Proteintech, 67763-1-Ig), anti-SLC7A11 (1:1000, Proteintech, 26864-1-AP), or anti-FTH1 (1:1000, Abcam, ab65080), followed by the addition of the corresponding secondary antibodies. The signal was detected using a chemiluminescence imaging system (Tanon 5200 Multi 4600SF, Shanghai, China).

### 2.8. Reverse Transcription-Quantitative PCR (RT-qPCR)

The purified DNA finally produced by CHIP was used as template DNA for the PCR reaction. qPCR analysis was performed using SYBR-Green I (Takara Bio, Inc., Kusatsu, Japan). GAPDH was used as an endogenous control. The primers used in this study were provided by Realgene (Realgene, Nanjing, China) (Supplementary Table S2): IDH1 forward, 5’-AGGTGGGCTGAGGAGGC-3’ and reverse, 5’-ACAGGGTAGGTCCGAGCTTT-3’; GAPDH forward, 5’-GAAGGTGAAGGTCGGAGTC-3’ and reverse, 5’-GAAGATGGTGATGGGATTTC-3’. Finally, the period threshold (Ct) value was analyzed by the 2^−ΔΔCt^ method. Each individual experiment was performed a total of three times.

### 2.9. Measurement of Intracellular GSH Level

RAW264.7 macrophages were seeded in 96-well plates (3 × 10^3^ cells/well) and treated with several experimental treatments 24 h after cell adhesion. After stimulation, a total glutathione detection kit (Beyotime, S0052, Shanghai, China) was used to detect the intracellular glutathione levels, and process the data, according to the manufacturer’s instructions. A microplate reader (Multiskan FC, Waltham, MA, USA) was used to measure the absorbance at 412 nm.

### 2.10. Iron Assay

RAW264.7 macrophages were seeded in 96-well plates (3 × 10^3^ cells/well) and treated with several experimental treatments 24 h after cell adhesion. After stimulation, an iron ion colorimetric detection kit (E1042, Applygen, Beijing, China) was used to analyze the iron concentration in each group. A microplate reader (Multiskan FC, Waltham, MA, USA) was used to measure the absorbance at 550 nm.

### 2.11. Lipid Peroxidation Assay

RAW264.7 macrophages were seeded in 96-well plates (3 × 10^3^ cells/well) and treated with several experimental treatments 24 h after cell adhesion. After stimulation, a lipid peroxide test kit (A106-1, Jiancheng, Nanjing, China) was used to evaluate the amount of lipid peroxide in each group. A microplate reader (Multiskan FC, Waltham, MA, USA) was used to measure the absorbance at 586 nm.

RAW264.7 macrophages were seeded in 6-well plates (1 × 10^6^ cells/well) and treated with several experimental treatments 24 h after cell adhesion. Lipid peroxidation levels were detected using a C11BODIPY probe (D3861, Thermo Fisher Scientific, Waltham, MA, USA). Cells were incubated with 2 μM C11BODIPY for 30 min at 37 °C, then washed three times with PBS. Finally, cells were imaged using a fluorescence microscope (THUNDER DMi8, LEICA, Munich, Germany). The mean fluorescence intensity of C11BODIPY Green, which measures the amount of lipid ROS in the cell, was calculated using ImageJ software (NIH, Bethesda, MD, USA).

### 2.12. Annexin V-APC/7-AAD Staining and Flow Cytometry

The Annexin V-APC/7-AAD apoptosis detection kit (KGA1025, KeyGen Biotech, Nanjing, China) was used to detect cell death. Cells were collected, dyes were applied, and the cells were treated for 20 min. Flow cytometry (Beckman Coulter Cytoflex S, Brea, CA, USA) was then used to examine the samples.

### 2.13. Oil Red O Staining

RAW264.7 macrophages were seeded in 6-well plates (1 × 10^6^ cells/well) and treated with several experimental treatments 24 h after cell adhesion, then washed three times with PBS and fixed in 4% paraformaldehyde for 30 min. We used oil red O to counterstain macrophages for an hour before being imaged under an optical microscope (Nikon TS2, Tokyo, Japan). Image J analysis software was further utilized to examine the experimental data.

### 2.14. LDH Release Assay

RAW264.7 macrophages were seeded in 6-well plates (1 × 10^6^ cells/well) and treated with several experimental treatments 24 h after cell adhesion. A commercially available lactate dehydrogenase assay kit (C0016, Beyotime, Shanghai, China) was used to detect LDH release. A microplate reader (Multiskan FC, Waltham, MA, USA) was used to measure the absorbance at 490 nm.

### 2.15. α-KG Assay

RAW264.7 macrophages were seeded in 6-well plates (1 × 10^6^ cells/well) and treated with several experimental treatments 24 h after cell adhesion. α-Ketoglutarate was collected using an α-ketoglutarate assay kit (MAK054, Sigma Aldrich, St. Louis, MO, USA), and a coupled enzyme assay was used to determine the α-ketoglutarate level. A microplate reader (Multiskan FC, Waltham, MA, USA) was used to measure the absorbance at 570 nm.

### 2.16. NADPH Assay

The NADPH/NADP ratio was determined using an NADP+/NADPH detection kit (WST-8 method) (S0179, Beyotime, Shanghai, China). Briefly, RAW264.7 macrophages (1 × 10^6^ cells) were split into two aliquots. One aliquot was left on ice (containing both NADP and NADPH), and the other was incubated at 60 °C for 30 min to deplete NADP (only NADPH remained). The content of NADPH was determined by the absorbance at 450 nm (Multiskan FC, Waltham, MA, USA). The concentrations of NADPH and NADP were calculated separately from the standard curve. Finally, we calculated the NADPH/NADP ratio.

### 2.17. JASPAR Analysis

The binding sites of IDH1 and NRF2 were predicted by the JASPAR database (accessed on 27 August 2021) (https://jaspar.genereg.net). The sequences 2000 bp upstream and 100 bp downstream of the origin of the IDH1 gene were obtained from the NCBI database as potential promoter regions (Chr2: 208254972-208257071). The sequences were then compared with the transcription factors in the JASPAR database to predict the possible binding sites of IDH1 and target transcription factors. Transcription factor binding sites (TFBS) in JASPAR are represented by visualization techniques. The ordinate represents the information content, and the greater the information content, the greater the probability of occurrence. The abscissa aligns the positions of TFBS, each position is formed by the stacking of possible bases [13].

### 2.18. Chromatin Immunoprecipitation (ChIP) Assay

Binding of IDH1 and NRF2 was detected using a chromatin immunoprecipitation (ChIP) assay kit (P2078, Beyotime, Shanghai, China). Briefly, different groups of RAW264.7 macrophages were treated with 37% paraformaldehyde. Lysates were sonicated and centrifuged. The supernatant was immunoprecipitated with the antibody anti-NRF2 overnight at 4 °C (1:100, 16396-1-AP, Proteintech). The same amount of mouse IgG antibody (1:200, cat. no. 554002, BD Biosciences, Franklin Lakes, NJ, USA) was used as a negative control. The DNA–protein complexes were eluted using elution buffer. Purified DNA was used as template DNA for PCR reactions.

### 2.19. Statistical Analysis

GraphPad Prism 8.0 was used for statistical analysis, and all experiments were conducted independently in triplicate. The data are presented in the form of mean ± standard deviation (SD). An analysis of variance was used to examine the mean differences between groups, with *p* values < 0.05 considered to be statistically significant.

## 3. Results

### 3.1. Identification and Enrichment Analysis of DEGs in Foam Cells

**Figure 1:**
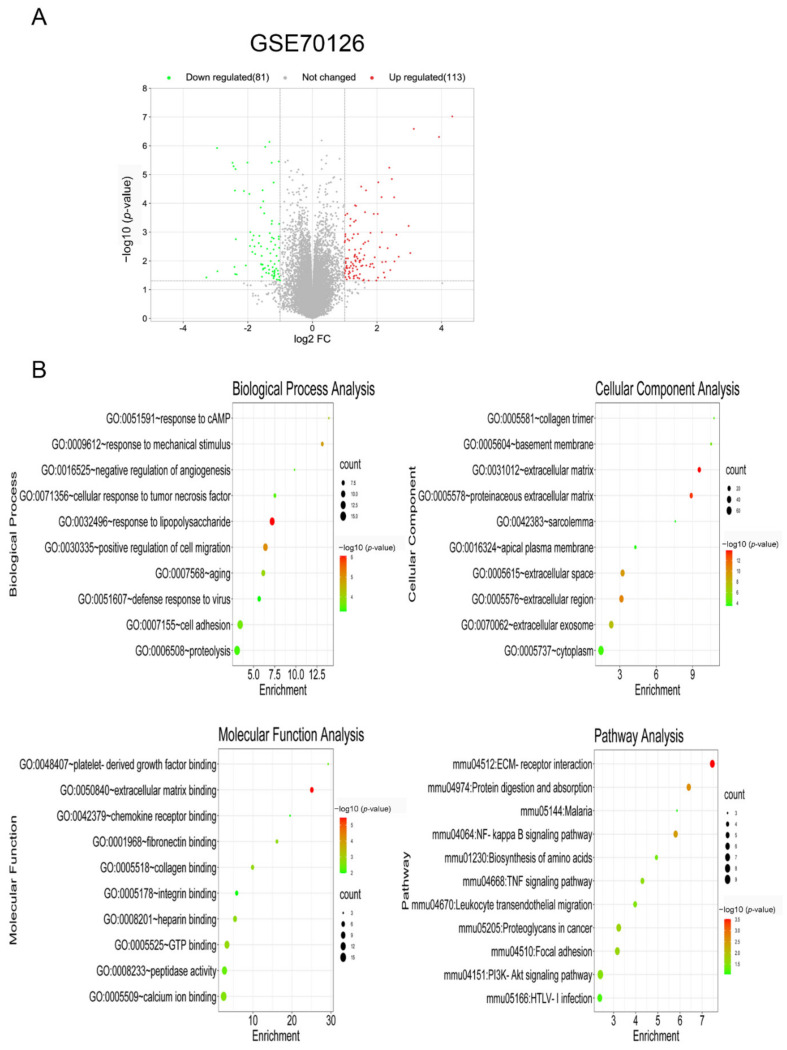
Identification and enrichment analysis of DEGs in foam cells. (**A**) Volcano plot of gene expression profile data between foam cells and macrophages in GSE70126. (**B**) Functional and signaling pathway analysis of DEGs between foam cells and macrophages.

**Figure 2:**
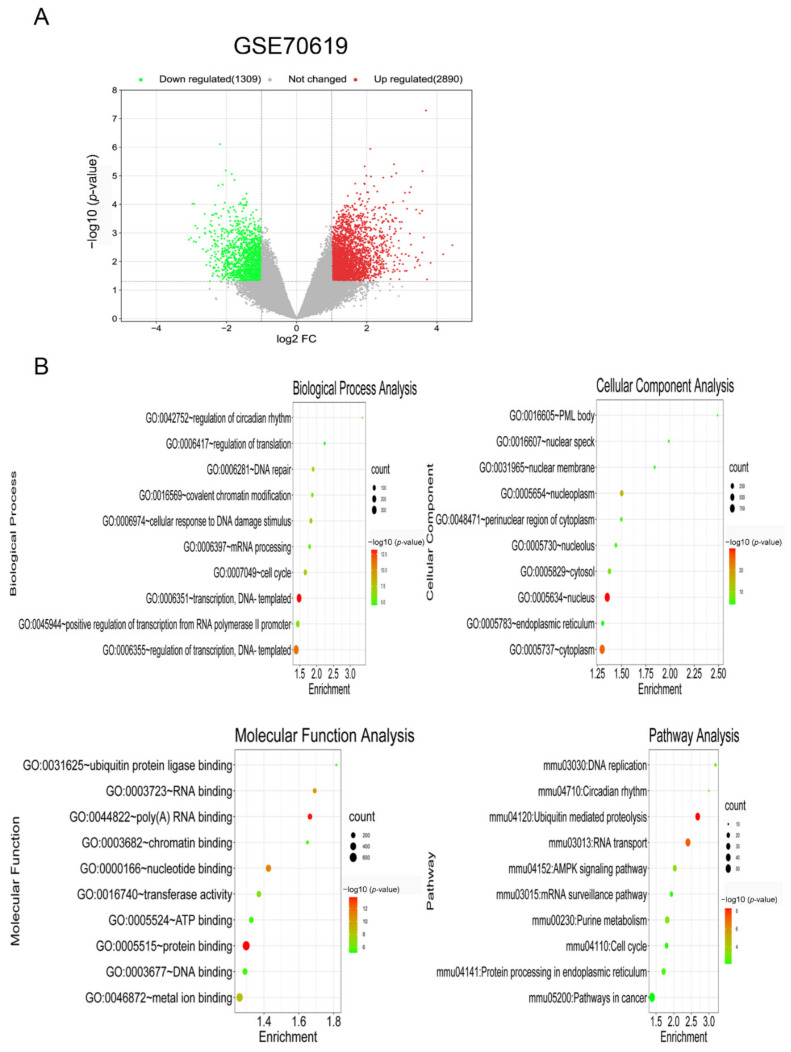
Identification and enrichment analysis of DEGs in foam cells. (**A**) Volcano plot of gene expression profile data between foam cells and macrophages in GSE70619. (**B**) Functional and signaling pathway analysis of DEGs between foam cells and macrophages.

Following GEO2R analysis of the GSE70126 and GSE70619 datasets, genes differentially expressed between foam cells and macrophages were discovered. Figure 1A and Figure 2A show the volcano map of DEGs in each dataset. To further understand the biological functions of the DEGs, we utilized DAVID to perform GO functional and KEGG pathway enrichment analyses on both datasets. The DEGs were substantially enriched in response to lipopolysaccharides and transcription, DNA-templated, according to GO analysis findings for biological processes (BP). The DEGs were found in the extracellular matrix and nucleus for cellular components (CC). The DEGs were considerably enriched in extracellular matrix binding and protein binding for molecular functions (MF). Furthermore, KEGG pathway analysis indicated that these DEGs were mostly engaged in ECM-receptor interactions, as well as ubiquitin-mediated proteolysis (Figure 1B and Figure 2B).

### 3.2. Identification and Enrichment Analysis of Target DEGs

**Figure 3:**
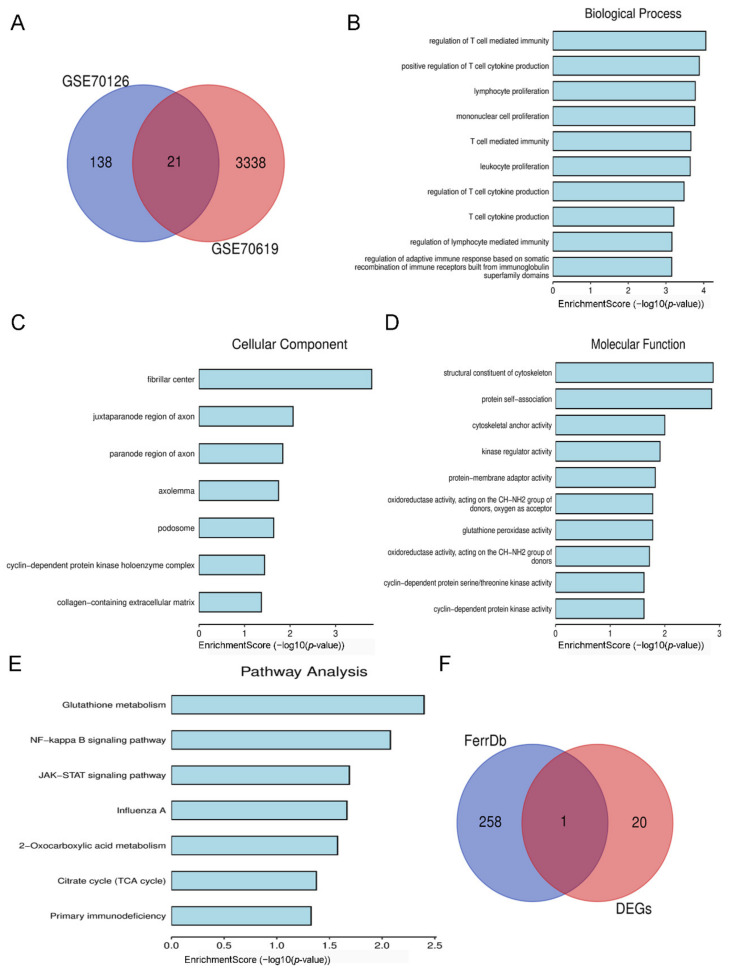
Identification and enrichment analysis of target DEGs. (**A**) Venn diagram of overlapping DEGs from GSE70126 and GSE70619 datasets. (**B**) Biological processes of 21 overlapping DEGs. (**C**) Cellular components of 21 overlapping DEGs. (**D**) Molecular functions of 21 overlapping DEGs. (**E**) KEGG pathway of 21 overlapping DEGs. (**F**) Venn diagram of 21 overlapping DEGs and 259 ferroptosis-related regulatory genes.

The Venn diagram revealed 21 DEGs that overlapped between the two datasets (Figure 3A), including 12 upregulated genes and 9 downregulated genes, for which we used Bioinformatics to undertake GO function and KEGG pathway enrichment analyses. A list of related genes is provided in the Supplementary Materials (Supplementary Table S1). For BP, 21 overlapping DEGs were considerably enriched in the regulation of T-cell-mediated immunity. A further 21 overlapping DEGs were considerably enriched in fibrillar centers in the case of CC; while for MF, 21 overlapping DEGs were enriched in structural constituents of cytoskeleton. In addition, these DEGs were predominantly involved in glutathione metabolism (Figure 3B–E). It is well known that ferroptosis is mainly manifested by decreased GSH and expression of GPX4; thus, we looked for differential genes associated with ferroptosis in overlapping DEGs as a target gene. After intersecting with the 259 ferroptosis-related regulatory genes sorted by FerrDb, we obtained a target gene IDH1 (Figure 3F).

### 3.3. Expression of IDH1 in ox-LDL-Induced Foam Cells

**Figure 4:**
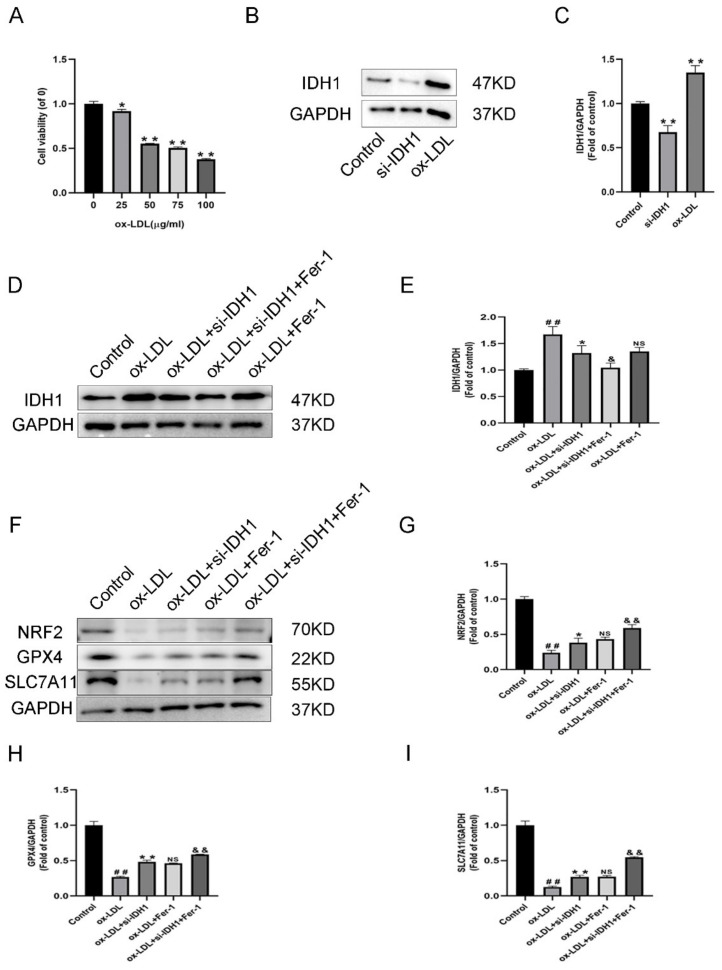
Expression of IDH1 in ox-LDL-induced foam cells. (**A**) Viability of RAW264.7 cells treated with ox-LDL (25, 50, 75, 100 µg/mL) for 24 h. * *p* < 0.05, ** *p* < 0.01 vs. control group. (**B**,**C**) Western blot was used to detect the levels of IDH1 protein expression. ^##^ *p* < 0.01 si-IDH1 treatment group vs. control group; * *p* < 0.05, ** *p* < 0.01 ox-LDL-treated group vs. control group. (**D**,**E**) Western blot was used to detect the levels of IDH1 protein expression. ^##^ *p* < 0.01 vs. control group; * *p* < 0.05, ** *p* < 0.01 vs. ox-LDL-treated group; ^&^ *p* < 0.05, ^&&^ *p* < 0.01 vs. ox-LDL and si-IDH1 treatment group. NS vs. ox-LDL and si-IDH1 treatment group. (**F**–**I**) NRF2, GPX4, and SLC7A11 expression. ^##^ *p* < 0.01 vs. control group; * *p* < 0.05, ** *p* < 0.01 vs. ox-LDL-treated group; NS vs. ox-LDL and si-IDH1 treatment group. ^&^ *p* < 0.05, ^&&^ *p* < 0.01 vs. ox-LDL and Fer-1 treatment group. NS, not significant. Data are presented as mean ± SD.

To develop a macrophage-derived foam cell model, different dosages of ox-LDL were used to stimulate RAW264.7 cells. In the CCK-8 experiment, 50 µg/mL ox-LDL significantly reduced cell viability (Figure 4A). As a result, in subsequent investigations, we used 50 µg/mL ox-LDL to enhance foam cell development, which was likewise the dosage used in previous findings [14,15].

In ox-LDL-induced foam cells, IDH1 was higher than in RAW264.7 cells, according to the Western blotting (WB) data (Figure 4B–E). The low IDH1 expression in the si-IDH1 group showed successful LV-Idh1-RNAi transfection (Figure 4B,C). Furthermore, for the ox-LDL + si-IDH1 group, low IDH1 expression could be successfully maintained. On the basis of IDH1 knockdown, Fer-1 was added, and the IDH1 protein level was further reduced. Interestingly, for IDH1 protein expression, the ox-LDL + si-IDH1 and ox-LDL + Fer-1 groups displayed no statistical difference. This suggests that the ferroptosis inhibitor Fer-1 and IDH1 knockdown have similar effects in reducing IDH1 expression.

### 3.4. Inhibition of IDH1 Can Reduce the Production of ox-LDL-Induced Foam Cells

The production of foam cells is a key step in the pathogenesis of atherosclerosis, and inhibiting foam cell formation could be a viable way to prevent atherosclerotic lesions from developing [2]. The oil red O staining findings showed that ox-LDL stimulation could enhance lipid accumulation, while the inhibition of IDH1 or addition of Fer-1 reduced the formation of foam cells. For ox-LDL + si-IDH1 + Fer-1 compared with ox-LDL + Fer-1, the experimental results showed that the formation of foam cells was further reduced, suggesting that the inhibition of IDH1 further ameliorated the formation of foam cells (Supplementary Figure S1A,B).

### 3.5. Inhibition of IDH1 Ameliorates ox-LDL-Induced Ferroptosis

Ferroptosis is dependent on the accumulation of intracellular iron causing an elevation of toxic LPO. In addition, it also revealed the decrease in GSH and the characteristic genes GPX4 and SLC7A11.

**Figure 5:**
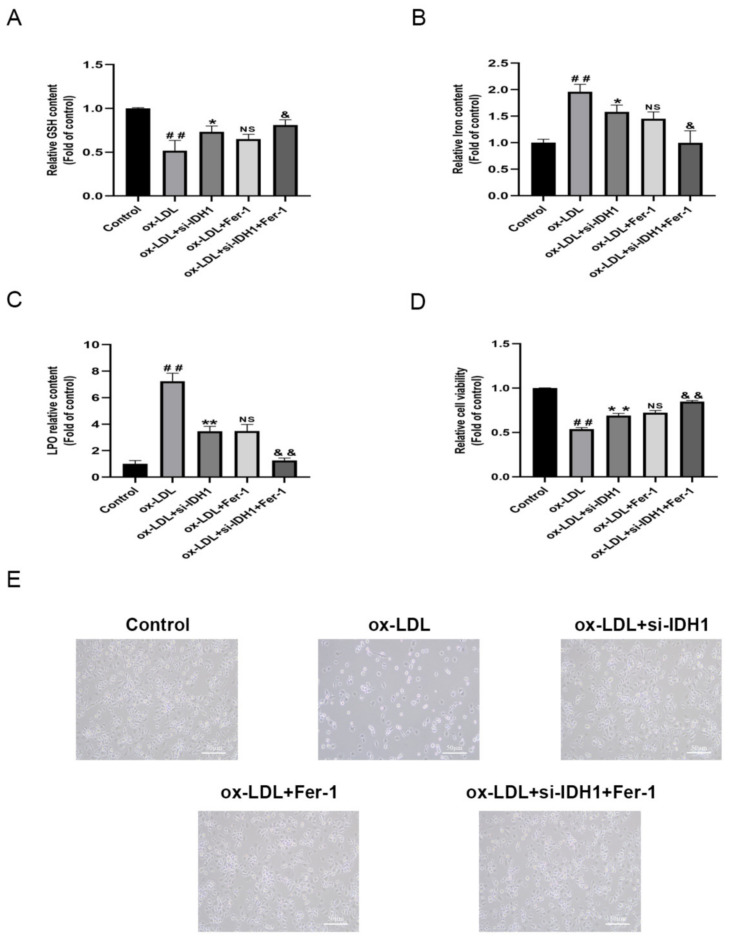
Inhibition of IDH1 ameliorates ox-LDL-induced ferroptosis. (**A**) The levels of GSH. (**B**) The level of iron content. (**C**) The total content of LPO. (**D**) Cell survival determined by CCK-8. (**E**) The brightfield images showing dead cells (Scale bars = 50 µm). ^##^ *p* < 0.01 vs. control group; * *p* < 0.05, ** *p* < 0.01 vs. ox-LDL-treated group; NS vs. ox-LDL and si-IDH1 treatment group. ^&^ *p* < 0.05, ^&&^ *p* < 0.01 vs. ox-LDL and Fer-1 treatment group. NS, not significant. Data are presented as mean ± SD.

RAW264.7 cells induced by ox-LDL showed higher Fe^2+^ levels, accumulation of LPO, depletion of GSH, and downregulation of GPX4, FTH1, and SLC7A11 protein levels, indicating that ox-LDL promotes macrophage ferroptosis (Figure 4F,H,I, Figure 5A–C and Figure S1C,D). The knockdown of IDH1 showed reduced Fe^2+^ and LPO accumulation and increased levels of GSH, GPX4, FTH1, and SLC7A11, implying that the inhibition of IDH1 could reduce the ferroptosis induced by ox-LDL (Figure 4F,H,I, Figure 5A–C and Figure S1C,D). The experimental results of the ox-LDL + si-IDH1 and ox-LDL + Fer-1 groups were similar, demonstrating that IDH1 inhibition can play a similar role in inhibiting ferroptosis as Fer-1 (Figure 4F,H,I, Figure 5A–C and Figure S1C,D). For ox-LDL + si-IDH1 + Fer-1 compared with ox-LDL + Fer-1, the IDH1 protein level was further reduced. The experimental results showed that the content of Fe^2+^ and LPO was further reduced, while the levels of GSH, GPX4, FTH1, and SLC7A11 were further increased, suggesting that the inhibition of IDH1 further ameliorated ox-LDL-induced ferroptosis (Figure 4F,H,I, Figure 5A–C and Figure S1C,D).

Lipid peroxidation was a critical biomarker associated with ferroptosis. We detected lipid peroxidation levels using C11BODIPY staining. The results showed that lipid peroxidation levels rose in ox-LDL-induced foam cells compared to the control group, but knocking down IDH1 reversed the increased lipid peroxidation levels. This suggested that ferroptosis was involved in ox-LDL-induced foam cell formation, and knocking down IDH1 relieved this by attenuating ferroptosis (Supplementary Figure S2A,B).

### 3.6. Inhibition of IDH1 Suppresses α-KG and NADPH Levels

It has been reported that the suppression of IDH1 inhibits α-KG and NADPH levels [16]. Therefore, we tested the α-KG and NADPH levels after generating the IDH1 KD macrophage cell line. The inhibition of IDH1 reduced the levels of α-KG and NADPH (Supplementary Figure S1E,F).

### 3.7. Inhibition of IDH1 Ameliorates Macrophage Damage and Apoptosis Induced by ox-LDL

**Figure 6:**
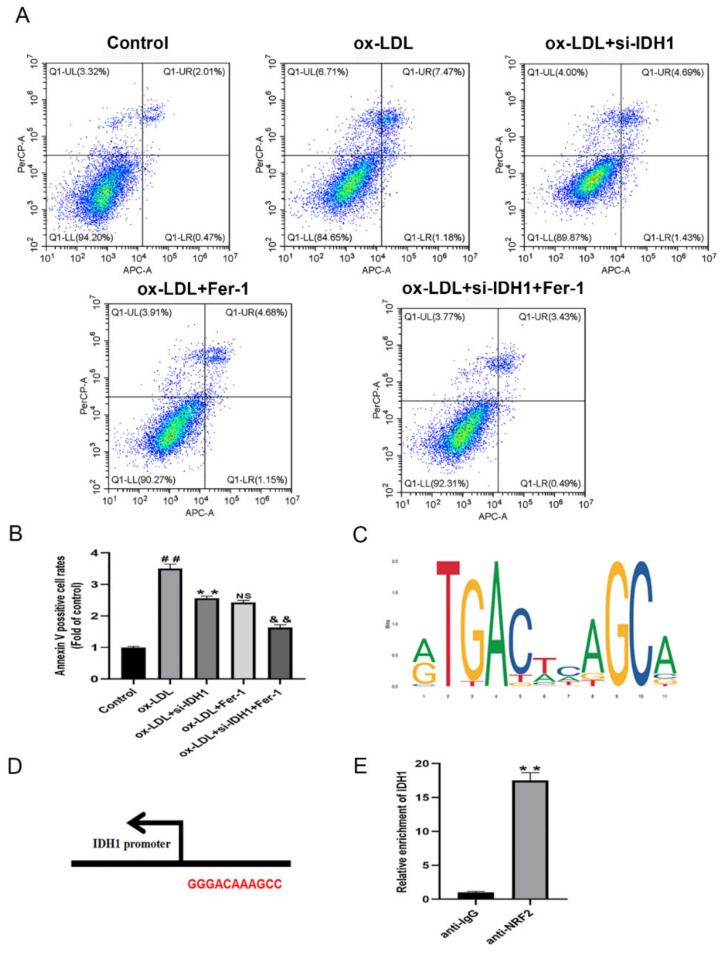
Inhibition of IDH1 ameliorates ox-LDL-induced macrophage apoptosis. (**A**,**B**) Apoptosis of RAW264.7 cells in different groups was examined via flow cytometry. (**C**) JASPAR-predicted NRF2 transcription factor binding sites. (**D**) Nrf2 and IDH1 potential binding sites. (**E**) Relative enrichment of IDH1, detected by ChIP. ^##^ *p* < 0.01 vs. control group; ** *p* < 0.01 vs. ox-LDL-treated group; NS vs. ox-LDL and si-IDH1 treatment group. ^&&^ *p* < 0.01 vs. ox-LDL and Fer-1 treatment group. NS, not significant. Data are presented as mean ± SD.

We used the CCK8 test to assess cell viability, and flow cytometry to quantify the amount of apoptosis to see how IDH1 affected ox-LDL-induced macrophage damage and apoptosis. Macrophage viability was lowered and apoptosis was increased when ox-LDL was added. However, the inhibition of IDH1 expression before ox-LDL induction improved macrophage viability and inhibited apoptosis. Between the ox-LDL + si-IDH1 and ox-LDL + Fer-1 groups, there was no significant change in the cell survival or apoptosis rate, implying that IDH1 downregulation served a comparable function to the ferroptosis inhibitor Fer-1. In addition, the inhibition of IDH1 expression on the basis of ox-LDL + Fer-1 further ameliorated ox-LDL-induced macrophage damage and apoptosis (Figure 5D,E and Figure 6A,B).

In addition, we complemented LDH experiments to investigate whether the knockdown of IDHI could protect macrophages from cell death at higher concentrations of ox-LDL (100 µg/mL). The experimental results showed that when ox-LDL was added, increased cell death resulted in increased LDH activity. However, the inhibition of IDH1 expression prior to ox-LDL induction suppressed LDH activity. The cellular levels of LDH did not change significantly between the ox-LDL + si-IDH1 and ox-LDL + Fer-1 groups, implying that IDH1 downregulation was functionally equivalent to the ferroptosis inhibitor Fer-1. Furthermore, the inhibition of IDH1 expression on the basis of ox-LDL + Fer-1 further ameliorated ox-LDL-induced macrophage damage. Therefore, we conclude that the knockdown of IDHI reduces macrophage death (Supplementary Figure S1G).

### 3.8. IDH1 May Regulate ox-LDL-Induced Macrophage Ferroptosis via NRF2

Ferroptosis is cell death caused by oxidative stress. By modulating gene expression in the GSH antioxidant system and iron metabolism, NRF2 can influence ferroptosis. Ferroptosis has been linked to cancer and neurological illnesses in a growing number of studies, and NRF2, which can regulate ferroptosis, is a promising pharmacological target. In our study, the online database JASPAR provided the TFBS of NRF2 (Figure 6C); the IDH1 and NRF2 potential binding sites are GGGACAAAGCC (Figure 6D). The presence of these binding sites on the IDH1 promoter suggests that IDH1 may interact with NRF2. The addition of ox-LDL reduced NRF2 levels when compared to controls, whereas the inhibition of IDH1 prior to ox-LDL induction upregulated the expression of NRF2, which was similar to the ox-LDL + Fer-1 group. In addition, further reducing the expression of IDH1 on the basis of ox-LDL + Fer-1 would further upregulate the protein level of NRF2 (Figure 4F,G). Further ChIP assays demonstrated that the IDH1 promoter sequence was significantly enriched by the anti-NRF2 antibody (Figure 6E). In summary, this means that IDH1 may regulate ox-LDL-induced macrophage ferroptosis and foam cell formation through NRF2.

## 4. Discussion

Foam cells are involved in all stages of atherosclerosis development [17]. The formation of macrophage-derived foam cells is caused by the dysregulation of lipid metabolism. When foam cells accumulate beneath the lipid-rich arterial endothelium, they begin to perform a variety of functions that promote the development of atherosclerosis, increasing the likelihood of plaque rupture [18]. Furthermore, foam cells do not exist in isolation and can contribute to the development of atherosclerosis through pro-inflammation of apoptotic cells. The formation of foam cells and apoptosis induced by ox-LDL are important in the pathogenesis of atherosclerosis [19]. After apoptosis and necrosis, foam cells die, and intracellular lipids accumulate on arterial walls, exacerbating atherosclerotic lesions [20]. Apoptotic cell removal has been shown to help with plaque stabilization [21]. Excess lipid accumulation in macrophages inhibits macrophage egress from plaques, resulting in macrophage apoptosis and secondary necrosis, which exacerbates foam cell formation and atherosclerotic progression [22]. Apoptosis of macrophages causes necrotic areas to enlarge in late-stage atherosclerotic plaques [23]. Numerous studies have shown that ox-LDL-induced macrophage apoptosis and foam cell formation play an important role in the onset and progression of atherosclerosis, providing information for clinical practice and drug development. The inhibition of ox-LDL-induced macrophage apoptosis and foam cell formation could be a potential treatment target for atherosclerosis [24,25].

Foam cells are essential to the formation of atherosclerosis lesions, and increasing data suggest that blocking foam cell formation is a possible treatment option [2,25]. In this study, we screened 21 potential target genes in foam cells through the GEO database. KEGG suggested that DEGs were mainly enriched in glutathione metabolism. The main manifestation of ferroptosis is the reduction in GSH and GPX4; thus, we evaluated the ferroptosis-related target gene IDH1 in 21 DEGs. In foam cells induced by ox-LDL, IDH1 was higher than in RAW264.7 cells. The inhibition of IDH1 ameliorated the foam cell formation induced by ox-LDL.

Ferroptosis is distinguished by the buildup of intracellular iron and the weakening of antioxidant ability, which results in programmed cell death [26]. Many pathogenic processes, including neurological diseases, myocardial I/R damage, and COVID-19 cardiac injury, have been linked to ferroptosis [27,28,29]. Existing studies have certified that ferroptosis is related to the progression of atherosclerosis [30,31,32]. IDH1 has the ability to convert isocitrate to oxalosuccinate, which is then converted to α-ketoglutarate. Tumor-derived IDH1 mutations promote lipid ROS accumulation and subsequent ferroptosis. The inhibition of IDH1 reduced the levels of α-KG and NADPH, as in previous reports [16].

IDH1 mutations decrease GPX4, a key enzyme in the ferroptosis and removal of lipid ROS, and accelerate glutathione depletion [10]. However, it is unclear whether IDH1 reduces ox-LDL-induced ferroptosis and, as a result, influences the progression of atherosclerosis. IDH1 inhibition increased the GPX4, FTH1, and SLC7A11 protein levels and repressed glutathione depletion, whereas downregulation of IDH1 decreased Fe^2+^, LDH levels, and lipid ROS generation. This means that the inhibition of IDH1 ameliorated ox-LDL-induced ferroptosis.

Ox-LDL-induced macrophage damage and apoptosis are critical in the formation and development of atherosclerosis [33,34], and inhibiting IDH1 expression in macrophages prevented viability and apoptosis death in our work. By increasing the generation of GPX4, GSH, and NADPH, and preserving cellular iron homeostasis, the NRF2-Keap1 pathway decreases atherosclerosis-related ferroptosis [30]. IDH1 has binding sites with NRF2, according to database predictions, and the inhibition of IDH1 could upregulate the expression of NRF2. It has been reported that the promoter region of IDH1 binds significantly to NRF2 [35]. Similarly, in our study, NRF2 can bind to the promoter region of IDH1. By activating NRF2, the inhibition of IDH1 may relieve foam cell formation by ameliorating ox-LDL-induced macrophage ferroptosis.

By influencing the gene expression of the GSH antioxidant system and iron metabolism, NRF2 can impact AS-related ferroptosis [30,36]. NRF2 is decreased in AS patients. In addition, when ox-LDL stimulates cells, NRF2 is significantly reduced [7]. In our study, ChIP showed that IDH1 was able to bind to NRF2. In addition, IDH1 may also affect the expression of NRF2 by regulating oxidative metabolism-related products. This indicates that IDH1 may regulate ox-LDL-induced macrophage ferroptosis through NRF2, and we will explore the relevant mechanism in subsequent studies.

We observed that ox-LDL induces macrophage ferroptosis, while ox-LDL promotes the upregulation of IDH1 expression. Given that IDH1 mutation can lead to iron death in cells as reported in previous studies [10,37], we speculate that there may be a regulatory relationship between IDH1 and ferroptosis. Moreover, we found that the knockdown of IDH1 expression with specific IDH1 siRNA ameliorated the reduction in GSH, iron accumulation, decreased cell viability, and increased lipotoxicity. After adding ferroptosis inhibitors, these phenomena were further improved, indicating that the inhibition of IDH1 could partially ameliorate ox-LDL-induced ferroptosis. However, ox-LDL-induced ferroptosis and IDH1 upregulation cannot indicate whether ferroptosis promotes increased IDH1 expression or IDH1 promotes ferroptosis, and these two phenomena may be parallel rather than causal. Although our present results show that IDH1 expression was further downregulated in the si-IDH1 + Fer-1 group compared to the si-IDH1 only group, there was no difference when Fer-1 was used alone. This suggested that the inhibition of ferroptosis will further downregulate IDH1 in the presence of low levels of IDH1. Ferroptosis was improved after the inhibition of IDH1, which may suggest that there may be a relationship between ferroptosis and IDH1-dependent positive feedback regulatory mechanisms. Therefore, in-depth studies are needed to clarify the regulatory relationship between ferroptosis and IDH1, such as the detection of IDH1 expression changes after the induction of ferroptosis with ferroptosis agonists. In addition, careful design is necessary to determine whether the overexpression of IDH1 promotes ferroptosis, while adding ferroptosis inhibitors at the same time reverses the ferroptosis caused by the overexpression of IDH1. More importantly, the regulatory mechanism was found through deeper exploration. This research will aid in a better understanding of IDH1’s numerous activities in ferroptosis and open the door for the discovery of new ferroptosis therapeutic targets.

For the ox-LDL + si-IDH1 + Fer-1 group, the phenotypes such as gene expression and cell death can only be partially rescued. Our study showed that neither the suppression of IDH1 levels nor the use of Fer-1 could completely alleviate ox-LDL stimulation-induced macrophage ferroptosis and foam cell formation. Similarly, adding Fer-1 on the basis of inhibiting IDH1 only partially alleviated the ferroptosis of macrophages and ox-LDL-induced foam cell formation, but could not completely reverse the cell damage caused by ox-LDL. In follow-up studies, we will try to enhance the efficiency of IDH1 intervention or increase the concentration of Fer-1 and explore the ratio between the two to explore whether ox-LDL stimulation-induced macrophage ferroptosis and foam cell formation can be completely rescued. The possible mechanism will be verified by deeper exploration.

Recent studies have discovered that Jak2V617F, ALOX5, and NCF2 are involved in the formation of atherosclerosis by regulating macrophage ferroptosis [38,39]. Furthermore, the important role of macrophage-mediated ferroptosis in cancer therapy has also been widely researched [40,41]. In colorectal cancer, there is a crosstalk between ferroptosis and three other programmed cell deaths, including necrosis, autophagy, and apoptosis [42]. An increasing number of ferroptosis inhibitors have been discovered in recent years [43], and ferroptosis has become a new therapeutic target for more disorders [44].

In our findings, knocking down IDH1 inhibited macrophage ferroptosis and foam cell formation, which is a potential research area for better understanding the occurrence and development of atherosclerosis. One of the study’s shortcomings is the lack of human or in vivo data, as well as investigations on non-macrophage-derived foam cells. GSE70126 and GSE70619 were from separate platforms, we examined their respective differential genes and then took the intersection to find similar differential genes. One of the study’s limitations might be the lack of batch effect correction. In further investigations, we will investigate the relevant mechanisms, potentially resulting in the discovery of novel treatment targets for ferroptosis.

## 5. Conclusions

Overall, in ox-LDL-induced macrophages, IDH1 was shown to be substantially expressed. The inhibition of IDH1 modulates the progression of atherosclerosis by ameliorating macrophage viability and apoptosis. Furthermore, inhibiting IDH1 may alleviate atherosclerosis by ameliorating ox-LDL-induced macrophage ferroptosis by activating NRF2.

## Data Availability

The original contributions presented in the study are included in the article/Supplementary Materials. Further inquiries can be directed to the corresponding authors.

## Supplementary Materials

The following supporting information can be downloaded at: https://www.mdpi.com/article/10.3390/biology11101392/s1, Figure S1: Oil red O staining and the levels of FTH1, α-KG, NADPH, and LDH; Figure S2: Measurement of lipid peroxidation; Table S1: Differentially expressed genes; Table S2: Primer sequences.
