# Supplementary material for: IDH1 Promotes Foam Cell Formation by Aggravating Macrophage Ferroptosis
Source: Biology (Basel). 2022 Sep 23;11(10):1392. doi: 10.3390/biology11101392 (PMC9598283; doi:10.3390/biology11101392)
Supplement: Supplementary file 1 [file biology-11-01392-s001.zip › biology-1842169-Supplementary/Supplementary Figure S1-2.pdf]

Supplemental Data File for IDH1 promotes foam cell formation by aggravating macrophage ferroptosis

This file includes:
Supplementary Figure S1-2

Supplementary Figure S1. Oil red O staining and the levels of FTH1, α-KG, NADPH and LDH

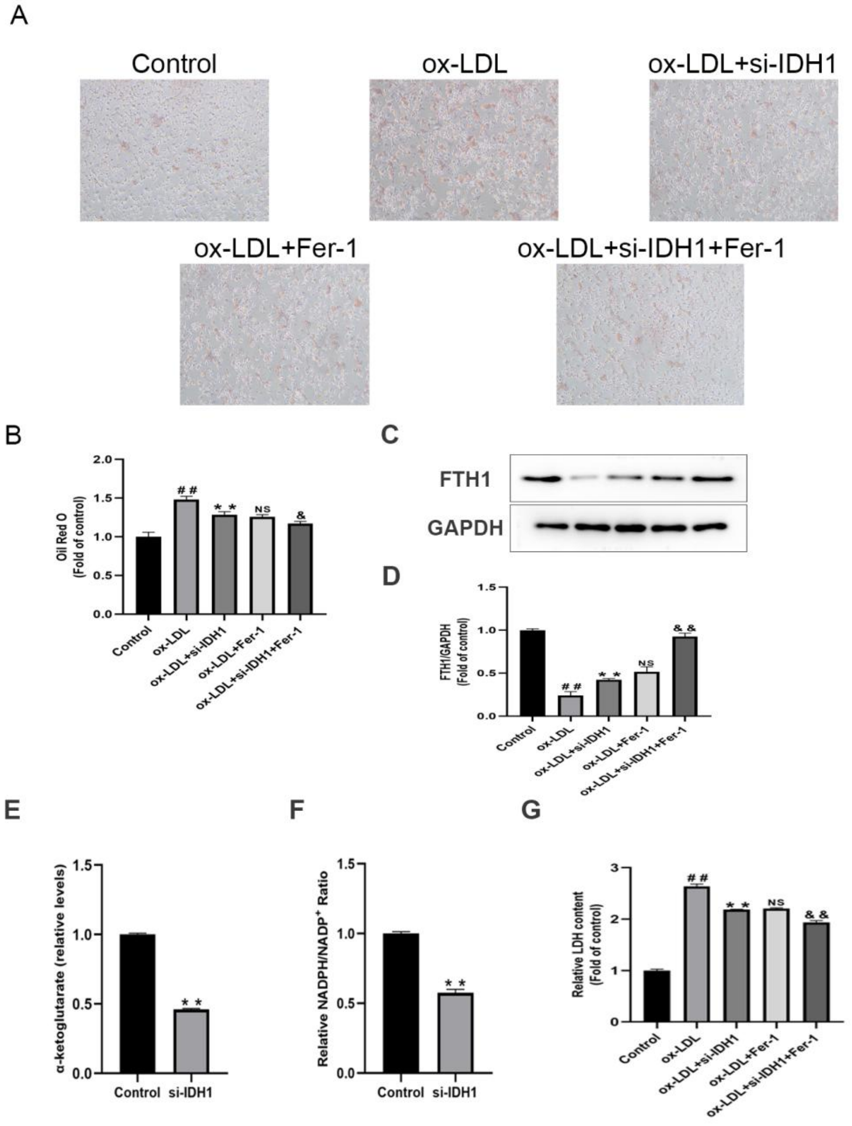

Supplementary Figure S1 (A-B) Oil red O staining. (**C-D**) Western blot was used to detect the levels of FTH1 protein expression. (**E**) The levels of α-KG. (**F**) The levels of NADPH. (**G**) The levels of LDH. $^{\#}P < 0.05$, $^{\#\#}P < 0.01$ vs. control group; $^{*}P < 0.05$,

**P < 0.01 vs. ox-LDL-treated group; NS vs. ox-LDL and si-IDH1 treatment group. &P < 0.05, &&P < 0.01 vs. ox-LDL and Fer-1 treatment group. Data are presented as mean±SD.

Supplementary Figure S2: Measurement of Lipid Peroxidation.

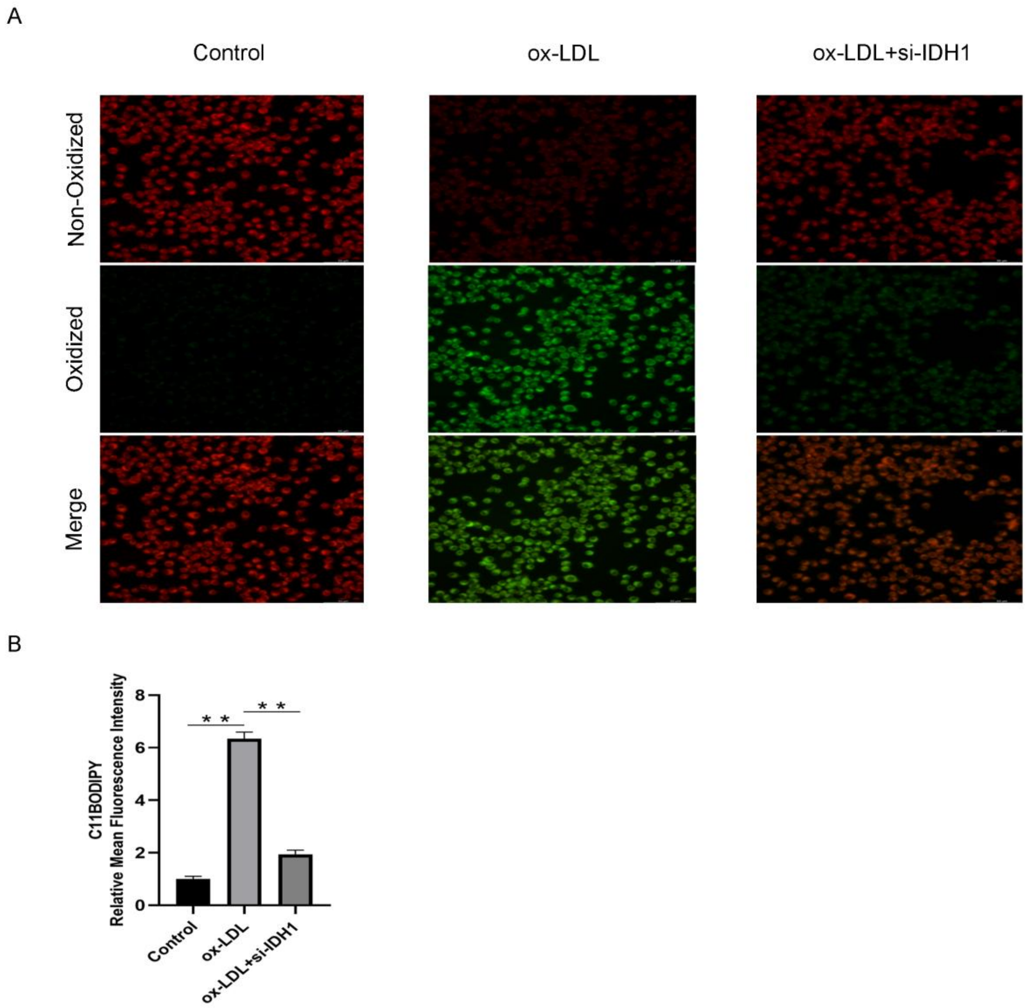

Supplementary Figure S2 (**A**) Representative images of C11BODIPY staining in different groups of cells. Scale bar=50μm. (**B**) The quantitative analysis of C11BODIPY relative mean fluorescence intensity. *P<0.05; **P < 0.01. Data are presented as mean ± SD.
